# 8-Hydroxyquinoline Dansylates Modified with PAMAM Dendrimer as Fluorescent Fe^3+ ^Sensors

**DOI:** 10.3390/molecules15052962

**Published:** 2010-04-27

**Authors:** Qi Zhang, Yaowu Sha, Jin-Hui Wang

**Affiliations:** 1 Department of Chemistry, The Key Laboratory of Bioorganic Phosphorous Chemistry and Chemical Biology, Tsinghua University, Beijing 100084, China; 2 School of Traditional Chinese Materia Medica, Shenyang Pharmaceutical University, Shenyang, China

**Keywords:** flourescent sensors, dendrimers, Fe^3+^ ion

## Abstract

A series of fluorescent sensors based on 8-hydroxyquinoline dansylate as core and dendritic PAMAM as shell were synthesized. Their fluorescence characteristic and fluorescent sensing behavior toward metal ions were studied in water/methanol (1: 1, *v/v*). The sensors exhibit good selectivity and sensitivity for Fe^3+^ ion and to some extent for Cr^3+^ ion. The third generation dendrimer has the better sensitivity than the first and second generations.

## 1. Introduction

Dendrimers are a class of macromolecules that have been attracting a lot of attention and extensively studied in recent years [1,2,3]. They have well-defined three-dimensional structures and different functional groups that allow the creation of molecules with desired properties. Nowadays, dendrimers are applied in different fields, ranging from materials and synthetic chemistry to biological and physical chemistry and so on [4,5,6,7,8,9,10,11].

Bonding a dye into the dendrimer structure contributes new properties and applications [12]. Dendrimers with fluorescent groups have found applications as components in different sensors. Also, the introduction of different types of chromophores to dendrimer macromolecules enables them to be photoactive with potential applications in photochemical molecular devices [13,14,15,16,17,18,19,20,21]. Some of these compounds have also been investigated for use as biosensors [22,23]. Over the years, many dendrimers with a fluorophore core have been synthesized in our laboratory. After dendritic modification, their fluorescent properties have been greatly altered [24,25,26,27].

Fe^3+^ ion plays important role in all living cells. It is present in the structure of many enzymes and proteins and therefore essential for cellular metabolism; however, exceeding concentrations of Fe^3+^ ion can also be detrimental. Thus, detection of Fe^3+^ ion is essential for monitoring the environment and human health. At present, there are only a few of Fe^3+^ ion fluorescence probes reported [28,29,30,31,32,33,34].

**Figure 1:**
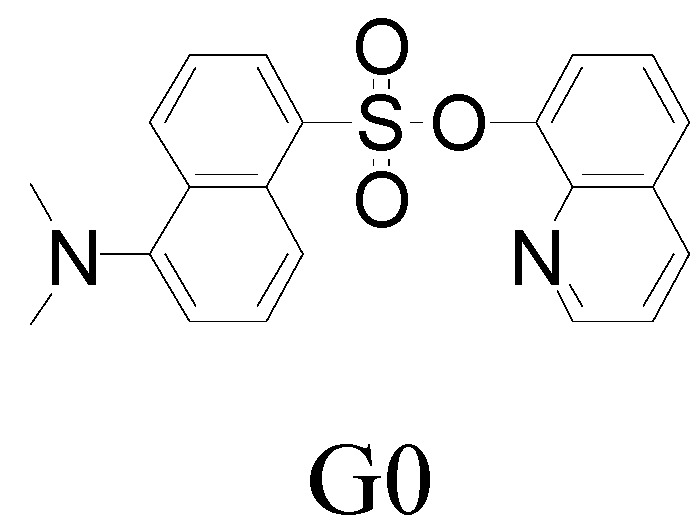
8-hydroxyquinoline dansylate of G0.

In our laboratory, 8-hydroxyquinoline dansylate of G0 (Figure 1) [35], as an effective Fe^3+^ ion fluorescent sensor, was synthesized. Its applications, however, were limited in three aspects: (a) it only dissolved in methanol, whereas many measurements were developed in water; (b) its low fluorescence sensitivity can rarely meet the requirement of detection in environment and biological fields; (c) as a Fe^3+^ ion fluorescence probe, it interfered with Cr^3+^ ion, and the quenching ratio of Fe^3+^/ Cr^3+^ was 1: 0.58. In this paper, three generations of dendritic fluorescent sensors based on 8-hydroxyquinoline dansylate as fluorophore core and PAMAM as shell were synthesized. With a number of amido linking to the dendrimer skeleton, their fluorescence sensitivity was enhanced and their water-solubility was improved significantly, which to some extent enlarged their applications in the measurement of Fe^3+^ ion. Furthermore, for G3.0, although Cr^3+^ ion still interfered with the measurement of Fe^3+^ ion, the quenching ratio of Fe^3+^/ Cr^3+^ was increased to 1: 0.50.

## 2. Results and Discussion

### 2.1. Synthesis of dendrimers

**Scheme 1:**
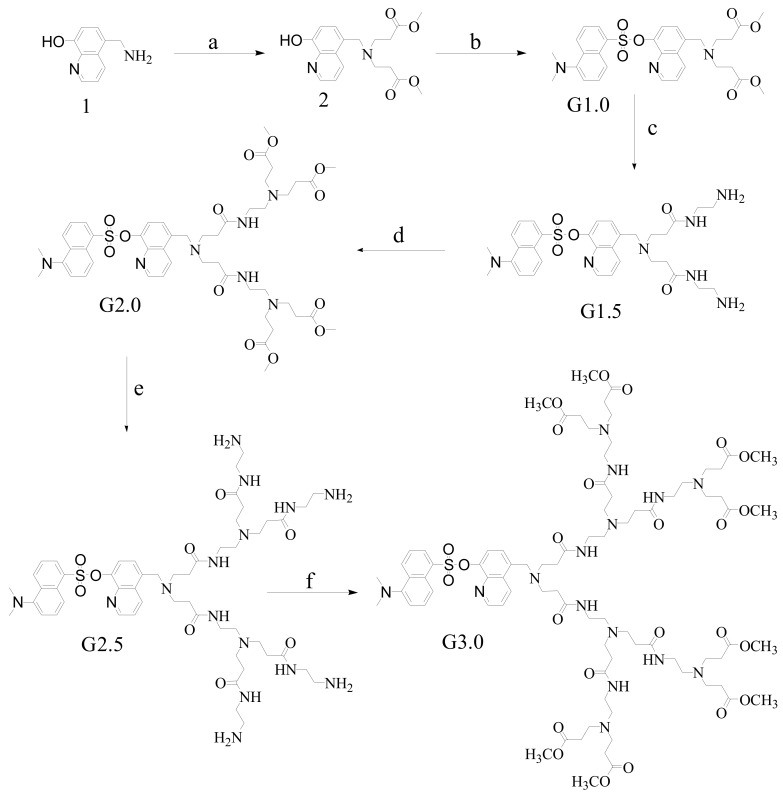
Synthesis of PAMAM dendrimers with 8-hydroxyquinoline dansylate as core.

The synthesis of G3.0 started from amine **1** and utilized six steps, as outlined in Scheme 1. Amine **1** was prepared by the Gabriel reaction according to the reported procedure [36]. Exhaustive Michael reaction of amine **1** with methyl acrylate was carried out in methanol to give diester **2**. The resulting diester **2** was then treated with 1.0 equiv of 1-(dimethylamino)-5-naphthalenesulfonyl chloride and 5.0 equiv of NaOH in TMF, to yield G1.0. The Michael reaction of G1.0 with a large excess of ethylenediamine was carried out in methanol to yield G1.5. The ester-terminated dendritic ligand G2.0 was prepared from G1.5 by the previous procedure. G2.5 and G3.0 were obtained through the previous PAMAM dendrimer iterative procedures. The structures have been identified by ^1^H-NMR, ^13^C-NMR, ESI-MS and elemental analysis.

### 2.2. Fluorescence emission properties

**Figure 2:**
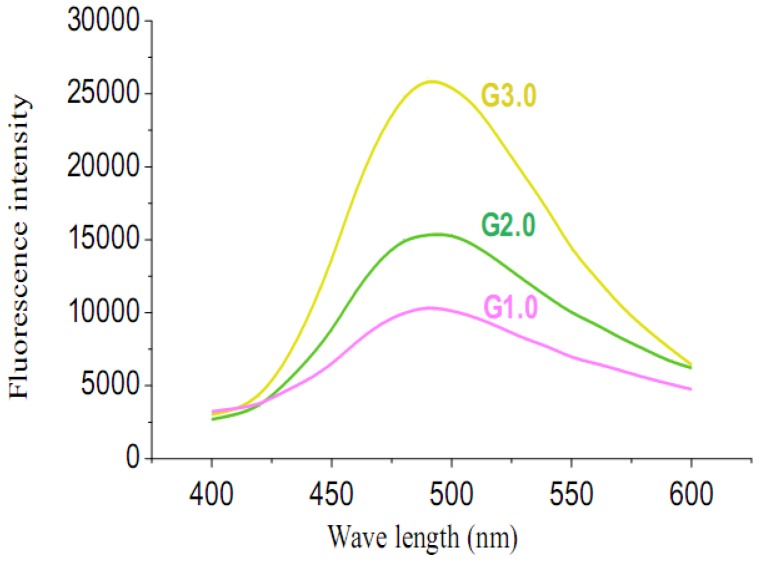
Fluorescent spectra of 5×10^-5^ mol/L G3.0, G2.0, G1.0 in H_2_O/CH_3_OH (1:1, *v/v*). Excitation wavelength is 320 nm. Emission wavelength is 490 nm.

In order to get clearer insight into the fluorescence of the different generations, the fluorescence behavior of each generation dendrimer was measured and shown in Figure 2. It is evident that the fluorescence intensity strengthened remarkably as the generation increased. The intensity of G3.0 and G2.0 was 2.5 times and 1.7 times greater than G1.0 respectively. With the increasing of the generation of the dendritic fluorescent sensors, their rate of self-quenching, as a result of site isolation of the chromophore, was reduced, which resulted in the remarkably improvement of the fluorescence efficiency and sensitivity.

**Figure 3:**
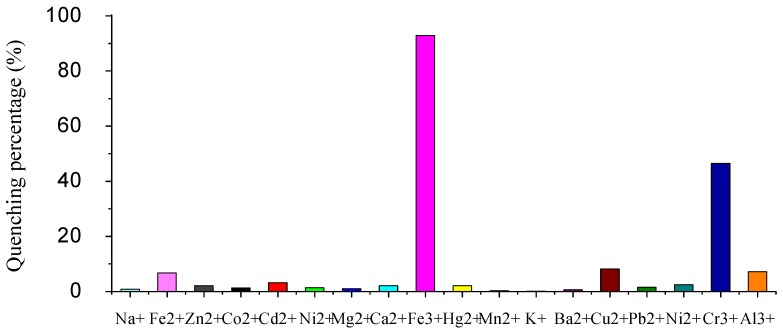
Quenching percentage [(I_0_-I)/I_0_] × 100% of fluorescence intensity of G3.0 (5 × 10^-5^ mol/L) upon the addition of 1.0 equiv metal ions in H_2_O/CH_3_OH (1:1, *v/v*). Excitation wavelength is 320 nm. Emission wavelength is 490 nm.

To obtain insight into the ability of G3.0 to sense selectively metal ions, we first investigated fluorescence changes upon the addition of various metal ions in H_2_O/CH_3_OH (1:1, *v/v*) and the results are shown in Figure 3. Without cations, G3.0 shows strong fluorescence. As Fe^3+^ ion was added to the solvent of G3.0, no shift of the fluorescence maximum was observed. The fluorescence, however, was almost quenched and remained 7.1% of its initial intensity. In contrast, addition of other metal cations (Na^+^, Fe^2+^, Zn^2+^, Co^2+^, Cd^2+^, Ni^2+^, Mg^2+^, Ca^2+^, Hg^2+^, Mn^2+^, K^+^, Ba^2+^, Cu^2+^, Pb^2+^, Ni^2+^and Al^3+^) scarcely showed fluorescence quenching, except for Cr^3+^, which has notable quenching. This phenomenon indicated a high selectivity in its fluorescence quenching response of G3.0 toward Fe^3+^ ion against other metal ions.

**Figure 4:**
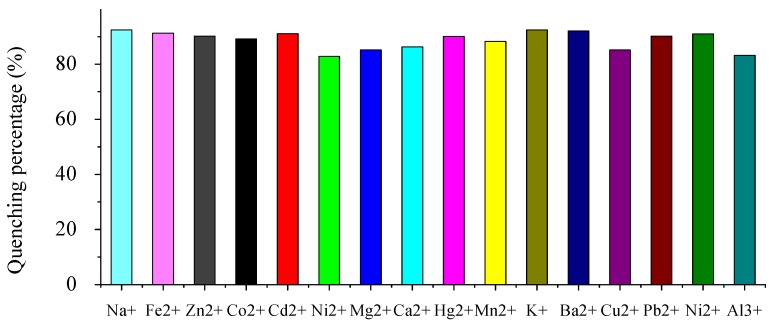
Fluorescence quenching percentage [(I_0_-I)/I_0_] × 100% of G3.0 (5 × 10^-5^ mol/L) upon the addition of 1.0 equiv Fe^3+^ ion and 10.0 equiv background in H_2_O/CH_3_OH (1:1, *v/v*). Excitation wavelength is 320 nm. Emission wavelength is 490 nm.

The selectivity and tolerance of G3.0 for Fe^3+^ ion over other metal ions was examined by competition experiments (Figure 4). When 1 equiv of Fe^3+^ ion was added into the solution of G3.0 in the presence of 10 equiv of other ions (Na^+^, Fe^2+^, Zn^2+^, Co^2+^, Cd^2+^, Ni^2+^, Mg^2+^, Ca^2+^, Hg^2+^, Mn^2+^, K^+^, Ba^2+^, Cu^2+^, Pb^2+^, Ni^2+^ and Al^3+^), respectively, the emission spectra displayed a similar quenching at near 490 nm to that of Fe^3+^ only. The excellent selectivity indicated that the fluorescence quenching by Fe^3+^ ion was scarcely affected by the co-existence of other metal ions. To further demonstrate the practical application of the probe, these metal ions were tested with higher concentrations, equivalent to the biological concentrations. Similarly, the fluorescence intensity of G3.0 in the presence of Fe^3+^ ion remained unchanged in the presence of other metals. This result clearly demonstrated the high selectivity of G3.0 towards Fe^3+^ ion, which is vital to investigate environmental and biological samples.

**Figure 5:**
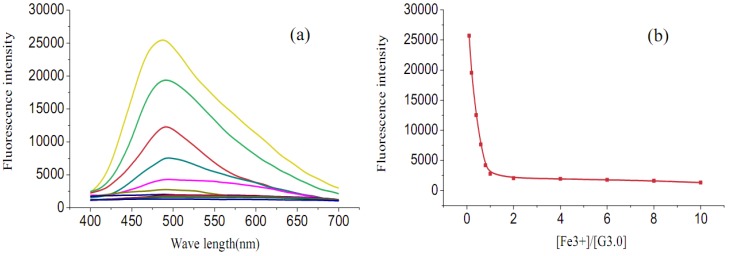
(a) The fluorescence spectra changes of G3.0 (5 × 10^-5^ mol/L) upon addition of Fe^3+^ ion in H_2_O/CH_3_OH (1:1, *v/v*); (b) The fluorescence spectra changes of G3.0 (5 × 10^-5^ mol/L) upon different ratio of [Fe3+]/[G3.0] in H_2_O/CH_3_OH (1:1, *v/v*).

The fluorescence spectra of G3.0 (5 × 10^-5^ mol/L) at various concentrations of Fe^3+^ ion are depicted in Figure 5a and 5b. As can be seen, the fluorescence intensity of G3.0 gradually decreased with the addition of Fe^3+^ ion. A well-defined titration break around 1 equiv of Fe^3+^ ion suggested a 1:1 stoichiometry of the G3.0-Fe^3+^ ion complex system [37].This phenomenon indicated that Fe^3+^ ion was coordinated with fluorophore core rather than the dendritic skeleton.

**Figure 6:**
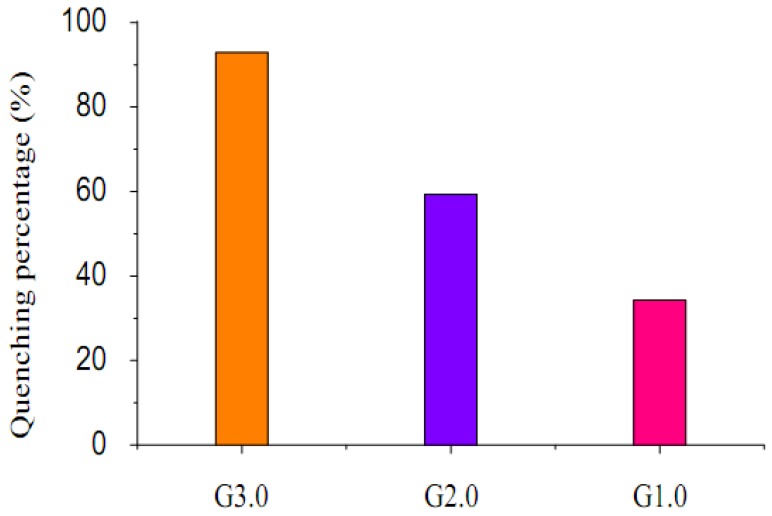
Fluorescence Quenching percentage [(I_0_-I)/I_0_] × 100% of G3.0, G2.0 and G1.0 (5 × 10^-5^ mol/L) upon the addition of 1.0 equiv Fe^3+^ ion in water/methanol (1:1, *v/v*). Excitation wavelength is 320 nm. Emission wavelength is 490 nm.

As the fluorescence intensity increased from G1.0 to G3.0, it is evident that the fluorescence quenching ratio to Fe^3+^ also increased. For G1.0, G2.0 and G3.0, these quenching ratio were 34.3%, 59.3% and 92.9% respectively, which was shown in Figure 6. This finding indicated that G3.0 behaved as a higher sensitive fluorescent Fe^3+^ ion sensor than G2.0 and G1.0.

## 3. Experimental

### 3.1. Materials and instruments

All solvents and reagents were purchased from Alfa Aesar, TCI, or Aldrich and used without further purification. ^1^H-NMR and ^13^C-NMR spectra were recorded as solutions in CDCl_3_ on a Jeol JNM ECA-300 (300 MHz) spectrometer with TMS as the internal standard. ESI-MS were recorded with a Perkin-Elmer ESQUIRE in the positive ion mode. Elemental analyses of carbon, hydrogen, and nitrogen were performed on a Carlo Erba-1106 microanalyzer. Fluorescence intensity was measured on a Bio-Tek synergy TM4.

### 3.2. Synthesis of G1.0

5-Aminomethyl-8-hydroxyquinoline (amine **1**) was prepared according to the reported procedure. Methyl acrylate (3.44 g, 40 mmol) was added to a solution of amine **1** (0.70 g, 4 mmol) in methanol (50 mL) at room temperature and the resulting mixture was stirred for 24 hours to give crude product of diester **2**. Then, the mixture was evaporated under vacuum and the crude product of diester **2** (0.69 g, 2 mmol) was dissolved in THF (50 mL). Sodium hydroxide (0.4 g, 10 mmol) was added in the solution and stirred for 5 minutes. Then a solution of dansyl chloride (0.54 g, 2 mmol) in THF (10 mL) was added drop wise at 0 °C in 10 minutes. The reaction was kept at room temperature for further 15 minutes and then filtrated. The filtrate was evaporated *in vacuo* and the crude product was purified by silica gel column chromatography (ethyl acetate:petroleum = 1:2). The product, G1.0, was obtained as yellow oil (64% yield). ^1^H-NMR (300 MHz, CDCl_3_): δ 8.74-8.71 (m, 2H), 8.60 (d, 1H, *J =* 8.61), 8.52 (d, 1H, *J =* 8.58), 8.18 (d, 1H, *J =* 7.20), 7.63 (t, 1H, *J =* 7.56), 7.46 (t, 1H, *J =* 7.20), 7.33–7.28 (m, 2H), 7.24–7.19 (m, 2H), 3.90 (s, 2H), 3.48 (s, 6H), 2.89 (s, 6H), 2.81 (m, 4H), 2.41 (m, 4H); ^13^C-NMR (75 MHz, CDCl_3_) δ172.5, ,151.5, 150.3, 145.5, 142.0, 134.2, 133.0, 132.3, 131.6, 130.5, 130.3, 129.7, 128.7, 128.6, 126.8, 122.8, 121.2, 120.8, 120.2, 115.4, 56.8, 51.4, 49.1, 45.4, 32.1; ESI-MS: calcd. for (M+H)/z: 580.2. Found: (M+H)/z: 580.3; Anal. calcd for C_30_H_33_N_3_O_7_S: C 62.16, H 5.74, N 7.25, S 5.53; Found C 62.09, H 5.81, N 7.29, S 5.42.

### 3.3. Synthesis of G2.0

Ethylenediamine (6.0 g, 100 mmol) was added to a solution of G1.0 (0.66 g, 1.1 mmol) in methanol (50 mL) at room temperature and the resulting mixture was stirred for 5 days to give crude product of G1.5. Then, the mixture was evaporated under vacuum and the crude product of G1.5 (0.83 g, 1.3 mmol) was stirred with methyl acrylate (1.29 g, 15 mmol) in methanol (50 mL) for two days at room temperature. The crude product was purified by silica gel column chromatography (ethyl acetate:methanol = 7:1) to yield a yellow oil, G2.0 (78% yield). ^1^H-NMR (300 MHz, CDCl_3_): δ 8.74–8.69 (m, 2H), 8.63-8.55 (m, 2H), 8.19 (d, 1H, *J =* 7.20), 7.64 (t, 1H, *J =* 7.56), 7.49 (t, 1H, *J =* 7.56), 7.40–7.33(m, 2H), 7.27–7.21 (m, 2H), 6.71 (s, 2H), 3.99 (s, 2H), 3.61 (s, 12H), 3.18–3.16 (m, 4H), 2.91–2.86 (m, 14H), 2.70–2.66 (m, 8H), 2.42–2.32 (m, 12H); ^13^C-NMR (75 MHz, CDCl_3_) δ173.6, 172.5, 151.6, 150.2, 145.6, 141.9, 134.2, 132.7, 132.3, 131.6, 130.5, 130.3, 129.7, 128.6, 128.5, 126.7, 122.3, 121.2, 120.8, 120.2, 115.8, 56.8, 52.7, 51.4, 49.9, 49.1, 45.4, 37.3, 33.2, 33.1; ESI-MS: calcd. for (M+H)/z: 980.4. Found: (M+H)/z: 980.4; Anal. Calcd. for C_48_H_65_N_7_O_13_S: C 58.82, H 6.68, N 10.00, S 3.27; Found C 58.99, H 6.78, N 10.29, S 3.48.

### 3.4. Synthesis of G3.0

Ethylenediamine (5.4 g, 90 mmol) was added to a solution of G2.0 (0.88 g, 0.9 mmol) in methanol (50 mL) at room temperature and the resulting mixture was stirred for seven days to give crude product of G2.5. Then, the mixture was evaporated under vacuum and the crude product of G2.5 (1.05 g, 0.96 mmol) was stirred with methyl acrylate (0.86 g, 10 mmol) in methanol (50 mL) for three days at room temperature. The crude product was purified by silica gel column chromatography (ethyl acetate:methanol = 4:1) to yield a yellow oil, G3.0 (76% yield). ^1^H-NMR (300 MHz, CDC_l3_): δ 8.75–8.73 (m, 1H), 8.69 (d, 1H, *J =* 8.58), 8.60 (t, 2H, *J =* 8.58), 8.20–8.17 (m, 1H), 787.67–7.61 (m, 1H), 7.53–7.47 (m, 3H), 7.42–7.38 (m, 2H), 7.25–7.17 (m, 2H), 7.00–6.97 (m, 4H), 3.99 (s, 2H), 3.64 (s, 24H), 3.25–3.17 (m, 12H), 2.91 (s, 6H), 2.78–2.70 (m, 28H), 2.50–2.31 (m, 40H); ^13^C-NMR (75 MHz, CDCl_3_) δ172.7, 172.0, 171.8, 151.3, 150.0, 145.0, 141.7, 134.5, 132.9, 132.1, 131.5, 130.2, 130.0, 129.5, 128.4, 128.3, 126.8, 122.6, 121.2, 120.7, 119.9, 115.3, 55.9, 53.3, 52.5, 52.1, 51.3, 49.6, 48.9, 45.2, 37.3, 36.8, 33.5, 33.1, 32.4; ESI-MS: calcd for (M+H)/z: 1781.1. Found: (M+H)/z: 1781.6; Anal. calcd for C_84_H_129_N_15_O_25_S: C 56.65, H 7.30, N 11.80, S 1.80; Found C 56.51, H 7.18, N 11.96, S 1.66.

## 4. Conclusions

A series of new PAMAM dendrimers with 8-hydroxyquinoine dansylate as the fluorescence core have been synthesized. As fluorescent sensors, with the increasing generation, their sensitivity and selectivity toward Fe^3+^ ion were remarkably improved. Furthermore, the water solubility of dendrimers was developed to some extent, which could dissolve in the mixture of water and methanol. 
